# Morin Attenuates Ovalbumin-Induced Airway Inflammation by Modulating Oxidative Stress-Responsive MAPK Signaling

**DOI:** 10.1155/2016/5843672

**Published:** 2015-12-13

**Authors:** Yuan Ma, Ai Ge, Wen Zhu, Ya-Nan Liu, Ning-Fei Ji, Wang-Jian Zha, Jia-Xiang Zhang, Xiao-Ning Zeng, Mao Huang

**Affiliations:** Department of Respiratory Medicine, The First Affiliated Hospital of Nanjing Medical University, 300 Guangzhou Road, Nanjing, Jiangsu 210029, China

## Abstract

Asthma is one of the most common inflammatory diseases characterized by airway hyperresponsiveness, inflammation, and remodeling. Morin, an active ingredient obtained from Moraceae plants, has been demonstrated to have promising anti-inflammatory activities in a range of disorders. However, its impacts on pulmonary diseases, particularly on asthma, have not been clarified. This study was designed to investigate whether morin alleviates airway inflammation in chronic asthma with an emphasis on oxidative stress modulation. *In vivo*, ovalbumin- (OVA-) sensitized mice were administered with morin or dexamethasone before challenge. Bronchoalveolar lavage fluid (BALF) and lung tissues were obtained to perform cell counts, histological analysis, and enzyme-linked immunosorbent assay. *In vitro*, human bronchial epithelial cells (BECs) were challenged by tumor necrosis factor alpha (TNF-*α*). The supernatant was collected for the detection of the proinflammatory proteins, and the cells were collected for reactive oxygen species (ROS)/mitogen-activated protein kinase (MAPK) evaluations. Severe inflammatory responses and remodeling were observed in the airways of the OVA-sensitized mice. Treatment with morin dramatically attenuated the extensive trafficking of inflammatory cells into the BALF and inhibited their infiltration around the respiratory tracts and vessels. Morin administration also significantly suppressed goblet cell hyperplasia and collagen deposition/fibrosis and dose-dependently inhibited the OVA-induced increases in IgE, TNF-*α*, interleukin- (IL-) 4, IL-13, matrix metalloproteinase-9, and malondialdehyde. In human BECs challenged by TNF-*α*, the levels of proteins such as eotaxin-1, monocyte chemoattractant protein-1, IL-8 and intercellular adhesion molecule-1, were consistently significantly decreased by morin. Western blotting and the 2′,7′-dichlorofluorescein assay revealed that the increases in intracellular ROS and MAPK phosphorylation were abolished by morin, implying that ROS/MAPK signaling contributes to the relief of airway inflammation. Our findings indicate for the first time that morin alleviates airway inflammation in chronic asthma, which probably occurs via the oxidative stress-responsive MAPK pathway, highlighting a novel profile of morin as a potent agent for asthma management.

## 1. Introduction

Allergic asthma, which is caused by inappropriate responses to inhaled allergens, is a heterogeneous inflammatory disorder characterized by airway hyperresponsiveness (AHR), remodeling, and inflammation [1]. Among these characteristics, chronic inflammation has attracted much attention for its contribution to asthma [2]. Conventional anti-inflammatory therapies such as glucocorticoids are merely ameliorative rather than curative and are associated with diverse unexpected side effects [3]. Some patients benefit little from these therapies and some even suffer from a series of adverse effects, including hyperglycemia, hyperlipidemia, hypertension, osteoporosis, and susceptibility to pathogens [4]. Thus, there is an urgent need for the safe and effective therapeutic options in asthma treatment.

As the first line of defense against challenges, bronchial epithelial cells (BECs) produce innate immune mediators that limit foreign antigen invasion, in addition to chemokines/cytokines that modulate immune responses under physiological conditions [5, 6]. During the development of asthma, which involves an aberrant airway immune response, insults such as infection, allergens, or environmental factors could alter the profile of BECs, initiating chronic inflammation by polarizing T-helper type 2 (Th2) lymphocytes and promoting the secretion of proinflammatory proteins, including eotaxin-1, monocyte chemoattractant protein-1 (MCP-1), interleukin- (IL-) 8, and intercellular adhesion molecule-1 (ICAM-1) [7–9]. BECs also contribute to airway remodeling by producing various extracellular matrix (ECM) proteins [10], which in turn affect inflammation, determining the outcome of asthma. Therefore, strategies targeting the modulation of BECs may represent a new option for asthma treatment.

Tumor necrosis factor alpha (TNF-*α*) is an important proinflammatory molecule secreted by both immunocytes and structural cells [11]. Accumulating data have shown that TNF-*α* is markedly increased during the process of asthma [12]. It elicits proinflammatory cytokines generation and evokes the activation of various cells, leading to an amplification of inflammatory responses [13]. Clinical trials of agents targeting TNF-*α* have been shown to be effective in asthma management [11]. Blockade of the activity of TNF-*α* notably decreases Th2 cytokines production, the serum IgE levels, and inflammatory cell infiltration [11, 14, 15]. This evidence highlights the critical role of TNF-*α* in the inflammation. Herein, we established an inflammatory model with TNF-*α in vitro*.

**Figure 1:**
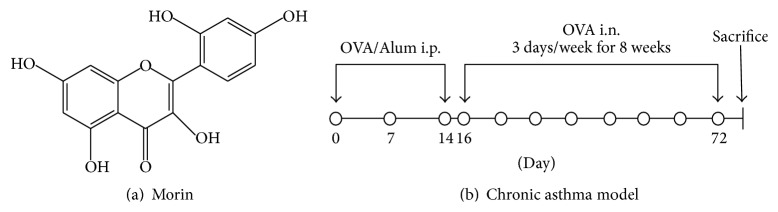
Chemical structure of morin and experimental protocol for the chronic asthma model. (a) Chemical structure of morin. (b) BALB/c mice were sensitized with OVA and aluminum hydroxide gel by intraperitoneal injection on days 0, 7, and 14 and then challenged with aerosolized 5% OVA for 30 min per day, three days per week for eight weeks, beginning on the 16th day of the experiment. The control mice were sensitized and challenged only with saline. Morin, DEX, or a vehicle (DMSO) was given by intraperitoneal injection at 30 min before each OVA challenge. i.p.: intraperitoneal injection; i.n.: inhalation.

Morin (3,5,7,2′,4′-pentahydroxyflavone), which exists in high concentrations in many herbs (Figure 1(a)), such as* Cudrania tricuspidata*, Osage orange,* Artocarpus heterophyllus* Lam., fig, and other Moraceae family members, has been shown to have strong antitumor and anti-inflammatory activities. Emerging data have indicated that morin protects rats from carbon tetrachloride-induced acute liver damage [16], suppresses the growth of hepatocellular carcinoma [17], and attenuates inflammatory responses in chronic experimental colitis [18]. Although morin has gained much attention in the treatment of a number of chronic diseases, it remains unclear whether it has benefits in asthma therapy. Given that asthma is characterized by airway inflammation and that morin has anti-inflammatory activities, the aim of the present study was to determine the impact of morin on allergic airway inflammation both* in vivo* and* in vitro*. The results obtained here indicate that morin significantly attenuates allergic airway inflammation, which might be due to an inhibition of reactive oxygen species (ROS)/mitogen-activated protein kinase (MAPK) signaling.

## 2. Materials and Methods

### 2.1. Animals

Specific pathogen-free female BALB/c mice (18–22 g) aged 6 to 8 weeks were obtained from Vital River Laboratories (Beijing, China). The mice were kept in a temperature-controlled room under a 12 h dark/light cycle and were provided with food and water* ad libitum*. All experiments that involved animal and tissue samples were performed in accordance with the guidelines of the National Institutes of Health and Nanjing Medical University, and all procedures were approved by the Institutional Animal Care and Use Committee of Nanjing Medical University (Nanjing, China).

### 2.2. Ovalbumin (OVA) Sensitization and Challenge

Figure 1(b) schematically depicts the protocols used in this study. In total, 42 specific pathogen-free female BABL/c mice were randomly divided into 6 groups as follows: control, OVA (Grade V, Sigma-Aldrich, Milwaukee, WI, USA), OVA + ML (5 mg/kg morin, Sigma), OVA + MH (10 mg/kg morin), OVA + dexamethasone (1 mg/kg DEX, Sigma), and OVA + dimethylsulfoxide (DMSO, Biosharp, Hefei, Anhui, China). The asthmatic models were established by sensitization to OVA. Specifically, all of the mice in the OVA, OVA + ML, OVA + MH, OVA + DEX, and OVA + DMSO groups were sensitized on days 0, 7, and 14 by intraperitoneal injection of 20 *μ*g OVA emulsified in 2 mg aluminum hydroxide gel (Invivo-Gen, San Diego, CA, USA) in a total volume of 200 *μ*L. These sensitized mice were exposed to aerosolized 5% OVA in sterile saline for 8 weeks beginning on the 16th day of the experiment, three times a week for 30 min each time. We placed the mice in 51 × 31 × 21 cm chambers that were connected to a jet nebulizer (NE-U11B; Omron Corp., Tokyo, Japan) to create a whole-body inhalation system. Morin (5 and 10 mg/kg), DEX (1 mg/kg, positive control), and DMSO (0.4 *μ*L in a total of 200 *μ*L saline, solvent control) were administered by intraperitoneal injection at 30 min before each OVA challenge. The control subjects were sensitized and challenged using the same protocol with saline alone. The mice were sacrificed at 24 h after the last challenge, and bronchoalveolar lavage fluid (BALF) and lung tissues were collected for analysis.

### 2.3. BALF Collection and Differential Cell Counts

Briefly, the mice were anesthetized by intraperitoneal injection of pentobarbital sodium (70 mg/kg) at 24 h after the final challenge. BALF was collected by lavage with ice-cold phosphate-buffered saline (PBS, 400 *μ*L × 3; 85–90% of the lavage volume was recovered) via a tracheal catheter. The lavage samples from each mouse were centrifuged at 1000 rpm for 10 min at 4°C. The total number of inflammatory cells in the BALF was counted using a hemocytometer. Differential cell counts were performed using Wright's staining on the basis of morphological criteria. The number of cells in the BALF was determined by two independent investigators in a single-blind study, and at least 200 cells each were analyzed from three different random locations using a microscope. Then, the supernatant was collected and divided into four equal portions and frozen at −80°C for enzyme-linked immunosorbent assay (ELISA).

### 2.4. Lung Histology

After BALF samples were collected, a 20 mL syringe equipped with a 18 G needle was used to inject 10–15 mL PBS slowly into the right ventricle. Then the lungs were inflated with 4% paraformaldehyde under 20 cm pressure by a tracheal catheter and placed in 4% paraformaldehyde fixative for paraffin embedding. A series of microsections (5 *μ*m) were cut with a microtome and stained with hematoxylin and eosin (H&E) to assess inflammatory cell infiltration. The inflammation score was determined as follows: grade 0: no inflammation; grade 1: occasional cuffing with inflammatory cells; and grades 2, 3, and 4: most bronchi or vessels which were surrounded by a thin layer (1-2 cells: grade 2), a moderate layer (3–5 cells: grade 3), or a thick layer (>5 cells: grade 4) of inflammatory cells, respectively. The total inflammation score was calculated by the addition of the peribronchial (PB) and perivascular (PV) inflammation scores. Periodic acid-Schiff (PAS) staining was used to quantify airway goblet cells, and Masson's trichrome staining was used to visualize collagen deposition and fibrosis. Both staining methods were scored as follows: 0: none; 1: <25%; 2: 25–50%; 3: 50–75%; and 4: >75% goblet cells [19–21]. Sections were also immunohistochemically stained for matrix metalloproteinase-9 (MMP-9). For the semiquantitative evaluation of MMP-9 expression, we used a scoring method modified by Sinicrope and Lu [22, 23]. The mean percentage of positive epithelial cells in the bronchi was determined in at least five areas at ×400 magnification and assigned to one of the following categories: 0: <5%; 1: 5–25%; 2: 25–50%; 3: 50–75%; and 4: >75%. The immunostaining intensity of MMP-9 was scored as 1+ (weak), 2+ (moderate), or 3+ (intense). The percentage of positive epithelial cells and the staining intensity were multiplied to produce a weighted score for each case. All of the scores were calculated by 2 independent observers who were blinded to the experiment, and at least three different fields were examined for each lung section.

### 2.5. Determination of Tissue Malondialdehyde (MDA) Level

The left lung tissues were homogenized on ice in normal saline. The homogenates were centrifuged at 4000 rpm at 4°C for 10 min. The MDA level in the supernatants was determined using the thiobarbituric acid reacting substances (TBARS) assay (Nanjing Jiancheng Corp., China) as previously described [23]. MDA reacts with thiobarbituric acid under acidic conditions at 95°C to form a pink-colored complex. This product can be measured at 532 nm. In this test, 1,3,3-tetraethoxypropane (TEP) was used as a standard.

### 2.6. Culturing and Morin Treatment of Normal Human BECs

Normal human BECs were purchased from the Beijing Institute for Cancer Research (Beijing, China). They were obtained from bronchial epithelial tissues of healthy adults who did not have a respiratory disease and did not smoke. The cells were cultured at 37°C and 5% CO_2_ in RPMI 1640 medium (Invitrogen-Gibco, Paisley, Scotland) supplemented with 20 U/L penicillin, 20 *μ*g/mL streptomycin, and 10% fetal bovine serum (Invitrogen-Gibco). Cells between passages 4 and 8 were used for the experiments. After serum starvation for 6–12 h, the cells were stimulated with 10 ng/mL TNF-*α* (Peprotech, Rocky Hill, USA) alone or in combination with morin (10 *μ*M), and they were further cultured for the indicated durations. Cells were treated in the same manner with N-acetylcysteine (NAC) as a positive control.

### 2.7. Cell Viability Assay

The cytotoxicity of morin on BECs was examined using the CCK-8 (Dojindo Molecular Technologies Inc., Kumamoto, Japan) assay. Human BECs were cultured in a 96-well plate at a density of 5 × 10^3^ cells per well and treated with morin at concentrations ranging from 0.1 to 200 *μ*M for 24 h. Then, CCK-8 solution was added to the cell culture medium at a 1 : 10 dilution, and the cultures were incubated for another 1-2 h at 37°C. Absorbance at 450 nm (A450) was measured with a microplate reader (CANY, Shanghai, China).

### 2.8. ELISA

To explore the effect of morin on TNF-*α*-induced inflammation in human BECs, human eotaxin-1, MCP-1, IL-8, and ICAM-1 (R&D Systems, Abingdon, UK) levels were measured. Cells were cultured using the aforementioned procedure and were then divided into the following four treatment groups: control, T (10 ng/mL TNF-*α*), T + M (10 ng/mL TNF-*α* + 10 *μ*M morin), and M (10 *μ*M morin). The cells were treated for 6 h as described above, and ELISAs were performed. The total IgE (Immuno-Biological Laboratories Co., Hamburg, Germany), TNF-*α*, IL-4, and IL-13 levels (R&D) in the BALF of the mice were also measured by ELISA, according to the manufacturer's instructions.

### 2.9. Determination of Intracellular ROS Production

Intracellular ROS were measured using the 2′,7′-dichlorofluorescin diacetate (DCFH-DA) assay. Briefly, 1.5 × 10^4^ cells were seeded into each well of a 6-well plate, cultured for 24 h, and exposed to morin (10 *μ*M) or NAC (10 mM) with TNF-*α* (10 ng/mL) for 6 h. The cells were then incubated with 10 *μ*M DCFH-DA for 30 min at 37°C in the dark. Next, they were washed twice with PBS and analyzed within 30 min using a FACScan instrument (Becton Dickinson, San Jose, CA, USA) with an excitation setting of 488 nm. The specific fluorescence signals corresponding to DCFH-DA were determined using a 525 nm band pass filter. For consistency, 10,000 cells were analyzed for each determination. Intracellular ROS production was also measured with a laser scanning confocal microscope (Zeiss LSM 5 live, German). After incubation with DCFH-DA, the cells were fixed with 4% paraformaldehyde for 10 min and washed three times with PBS before being photographed. The excitation and emission wavelengths used were identical to those described previously, and photographs were taken. For each culture, a minimum of 5 random fields were captured.

### 2.10. Western Blotting

Total cellular protein was collected following lysis in lysis buffer (Cell Signaling Technology Inc., Beverly, MA, USA) on ice and centrifugation for 15 min at 14,000 rpm at 4°C. The supernatant was transferred into a fresh tube and denatured in sodium dodecyl sulfate-polyacrylamide gel electrophoresis (SDS-PAGE) Loading Buffer (Beyotime, Shanghai, China) with heating to 100°C for 5 min, and it was then stored at −80°C. The total protein concentration was determined using the BCA protein assay (Thermo, Rockford, IL, USA). Proteins were separated by 10% SDS-PAGE. After electrophoresis, the separated proteins were transferred to polyvinylidene difluoride membranes (Millipore, Billerica, MA, USA) using the wet transfer method. Nonspecific sites were blocked with 5% nonfat milk in TBS Tween 20 (TBST; 25 mM Tris [pH 7.5], 150 mM NaCl, and 0.1% Tween 20) for 2 h, and the blots were incubated with primary antibodies (Cell Signaling Technology Inc.), including anti-glyceraldehyde-3-phosphate dehydrogenase (GAPDH), anti-phospho-p38, anti-p38, anti-phospho-ERK, anti-ERK, anti-phospho-JNK, and anti-JNK antibodies, overnight at 4°C. Goat anti-rabbit horseradish peroxidase-conjugated IgG (Cell Signaling Technology Inc.) was used to detect antibody binding. After treatment of the membranes with enhanced chemiluminescence system reagents (Thermo), the binding of specific antibodies was visualized using a Bio-Rad Gel Doc/Chemi Doc Imaging System and analyzed by Quantity One software.

### 2.11. Statistical Analysis

The data are expressed as the mean ± standard deviation (SD). All tests were performed using Prism 6.00 (GraphPad Software, San Diego, CA, USA) and SPSS version 20 (SPSS Inc., Chicago, IL, USA). The results were analyzed by one-way analysis of variance for repeated measures, followed by Dunnett's post hoc test to determine differences among multiple comparisons. The significance level was set to *P* < 0.05.

## 3. Results

### 3.1. Morin Attenuated Allergic Airway Inflammation in OVA-Sensitized Mice

**Figure 2:**
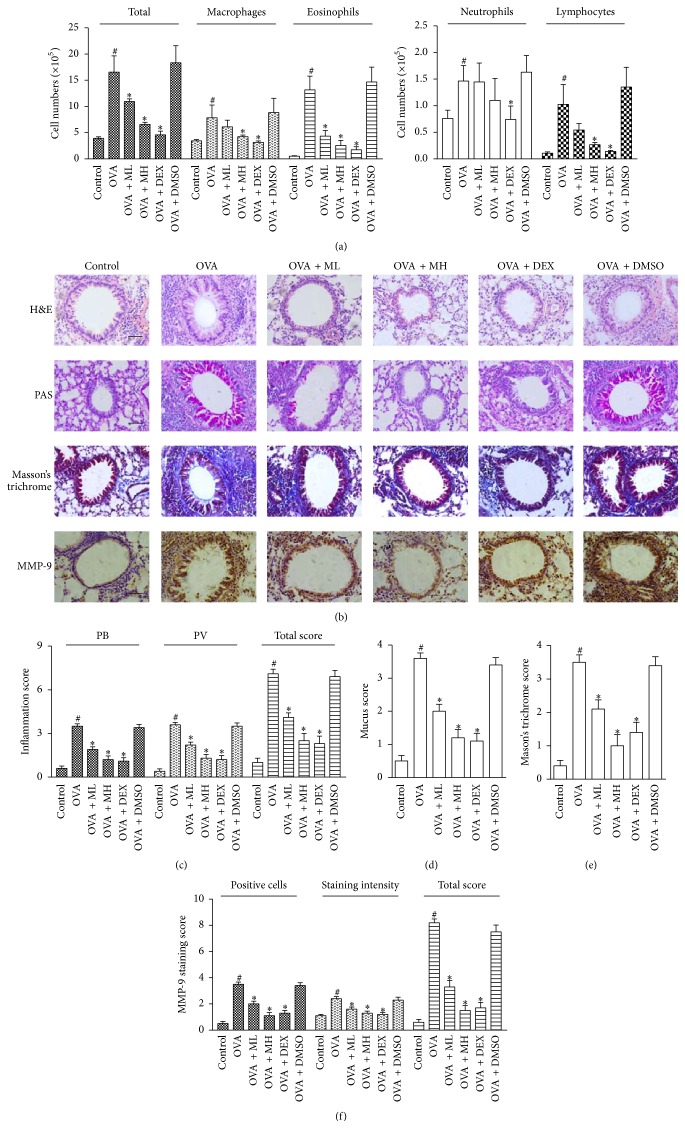
Treatment with morin reduced inflammatory cells infiltration, goblet cell hyperplasia, collagen deposition, and the expression of MMP-9 in lung tissue (magnification 400x). (a) Cell numbers and differentiation in BALF were determined by hemocytometer, and at least 200 cells were counted (*n* = 7 per group). (b) Lung sections were stained with H&E to analyze the infiltration of inflammatory cells, PAS to assess goblet cell hyperplasia, Masson's trichrome to evaluate the subepithelial deposition of collagen and fibrosis, and immunohistochemistry to assess the distribution of MMP-9. Scale bar: 50 *μ*m. (c) The layers of inflammation cells were counted and the total inflammation score was summed up with peribronchial (PB) and perivascular (PV) inflammation scores. (d) PAS-positive and PAS-negative epithelial cells were counted, and the percentage of PAS-positive cells per bronchiole was calculated. (e) Masson's trichrome staining analysis of collagen deposition was calculated. (f) The MMP-9 expression was evaluated and the total MMP-9 staining score was multiplied up with percentage of positive epithelial cells and staining intensity scores. Values represented as mean ± SD (*n* = 7 per group). ^#^ *P* < 0.05 compared with the control group, and ^*∗*^ *P* < 0.05 compared with the OVA group.

Lung sections were stained with H&E, and inflammatory cells in BALF were counted at 24 h after the last OVA challenge. Compared with the mice in the control group, those in the OVA and the vehicle group (OVA + DMSO) displayed severe airway inflammatory responses, including extensive infiltration of inflammatory cells into the BALF (Figure 2(a)) and around the respiratory tracts and vessels (Figure 2(b)). Treatment with morin or DEX suppressed the infiltration of inflammatory cells to varying degrees. Administration of morin (10 mg/kg) induced a remarkable decrease in not only the total cell counts but also the numbers of macrophages, eosinophils, and lymphocytes compared with those observed in the untreated asthmatic mice (*P* < 0.05), while the lower dose of morin (5 mg/kg) did not cause such drastic decreases in the cell numbers (Figure 2(a)). These results were further confirmed by H&E analysis and inflammation scores. Mice treated with morin (5 and 10 mg/kg) and DEX had fewer PB and PV inflammatory cells (Figure 2(b)), and the total inflammation scores were 4.1 ± 0.99, 2.5 ± 1.58, and 2.3 ± 1.64, respectively (*P* < 0.05) (Figure 2(c)). All of these findings indicated that administration of morin before the OVA aerosol challenge dose-dependently attenuated the inflammatory responses in the asthmatic airways.

### 3.2. Morin Abrogated Goblet Cell Hyperplasia in OVA-Sensitized Mice

The number of goblet cells and the extent of mucus production were assessed by PAS staining, and the percentage of PAS-positive cells in the bronchioles was also evaluated. We observed that the OVA-challenged mice developed marked goblet cell hyperplasia and mucus hypersecretion in the lumens of the bronchioles (Figure 2(b)). The morin- (10 mg/kg) and DEX-treated animals had fewer goblet cells in the airway epithelium, and the mucus scores in these two groups were reduced to 1.2 ± 0.79 and 1.1 ± 0.74 (*P* < 0.05), respectively, indicating the equivalent effects of the treatments (Figure 2(d)).

### 3.3. Morin Impaired Collagen Deposition/Fibrosis in OVA-Sensitized Mice

The area of collagen deposition/fibrosis was assessed using Masson's trichrome staining. Collagen deposition was profoundly enhanced in the interstitia of the airways and vessels of the tissues in the OVA group mice compared with the control group mice. Airway fibrosis was significantly ameliorated by administration of 10 mg/kg morin, with a score of 1.0 ± 1.05 (*P* < 0.05). The OVA + DEX group mice also showed significantly less fibrosis than the untreated asthmatic mice. However, no significant reduction in collagen deposition was observed in the OVA + ML group mice (Figures 2(b) and 2(e)).

### 3.4. Morin Decreased Expression of MMP-9 in OVA-Sensitized Mice

Representative photomicrographs of immunohistochemical staining for MMP-9 in the airways are shown in Figure 2(b). The densities of MMP-9 staining around the bronchioles and the infiltrated inflammatory cells in the OVA-challenged mice were higher than those in the control mice, and the score was 8.2 ± 0.92 (*P* < 0.05). These increases were dramatically reversed by the administration of DEX or the high dose of morin, with scores of 1.7 ± 1.34 and 1.5 ± 1.18, respectively (*P* < 0.05) (Figure 2(f)).

### 3.5. Morin Reduced Levels of IgE, TNF-*α*, and Th2 Cytokines in BALF

**Figure 3:**
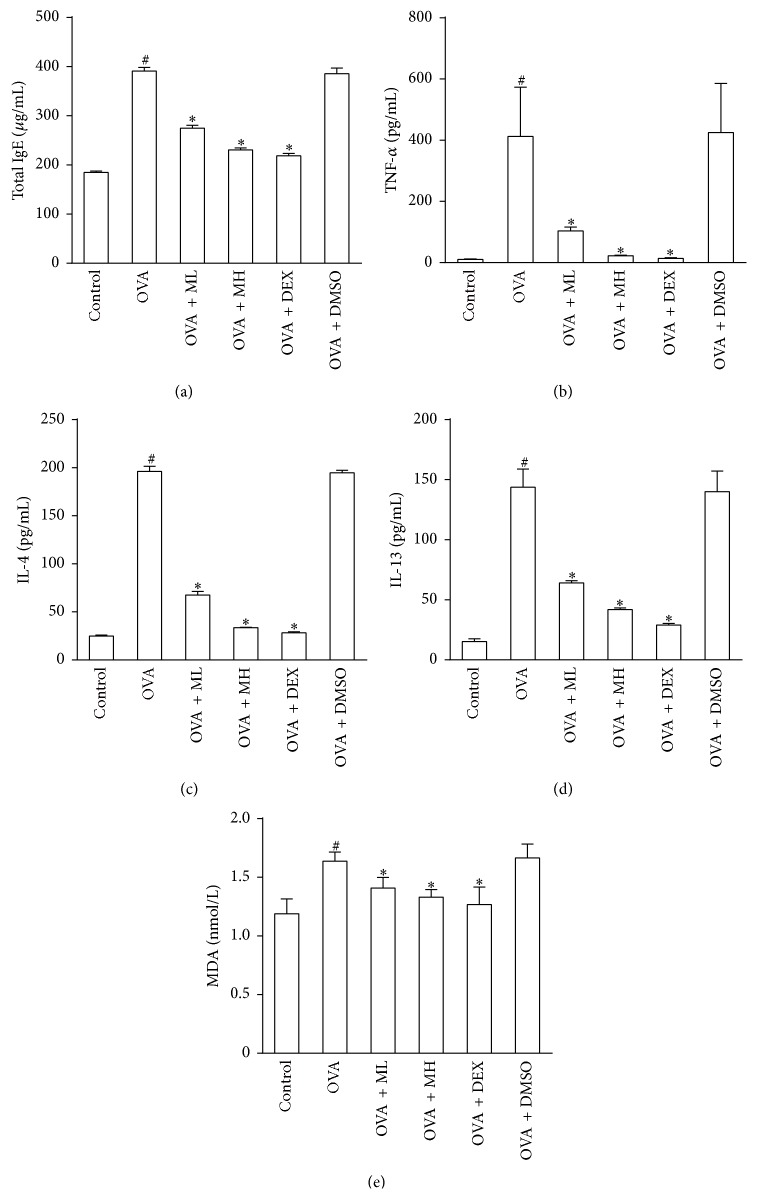
Treatment with morin inhibited the levels of IgE, TNF-*α*, and Th2 cytokines in BALF and MDA in lung tissues. (a–d) The concentrations of IgE, TNF-*α*, IL-4, IL-13, and MDA were measured with ELISA. Values represented as mean ± SD (*n* = 7 per group). ^#^ *P* < 0.05 compared with the control group, and ^*∗*^ *P* < 0.05 compared with the OVA group.

The total IgE, TNF-*α*, IL-4, and IL-13 levels in the BALF were notably increased by airway challenge with OVA. Administration of morin dose-dependently reduced the levels of IgE, Th2 cytokines, and TNF-*α* in the BALF compared with those in the BALF of the OVA group mice (*P* < 0.05) (Figures 3(a)–3(d)). These findings indicated that morin could inhibit allergic airway reactions by modifying Th2-predominant immune activity in the OVA-induced mouse asthma model.

### 3.6. Morin Inhibited MDA Level in Lung Tissues

To determine whether morin inhibits OVA-induced airway inflammation by the scavenging of free radicals, we detected the MDA level in the lung tissues to evaluate the changes in OVA-induced oxidative damage. As shown in Figure 3(e), the concentrations of MDA in the lung tissues in the OVA and vehicle groups (1.638 ± 0.17 nmol/L and 1.666 ± 0.20 nmol/L, resp.) were significantly higher than that in the control group (1.189 ± 0.25 nmol/L) (*P* < 0.05). The MDA levels in the lung tissues in the DEX and morin (10 mg/kg) pretreatment groups (1.267 ± 0.21 nmol/L and 1.330 ± 0.09 nmol/L, resp.) were significantly decreased compared with that in the OVA group (*P* < 0.05).

### 3.7. Morin Restrained TNF-*α*-Induced Proinflammatory Protein Expression in Human BECs

**Figure 4:**
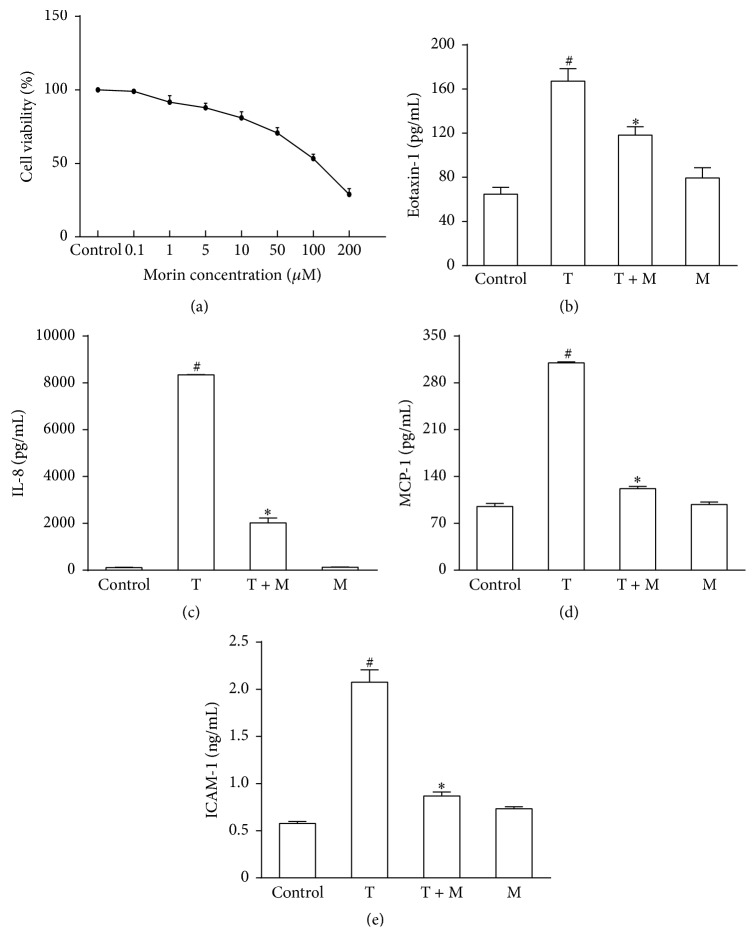
Treatment with morin restrained the proinflammatory proteins induced by TNF-*α* in BECs. (a) Effects of morin on the viability of human BECs assessed by CCK-8. (b–d) The expression levels of MCP-1 (b), eotaxin-1 (c), IL-8 (d), and ICAM-1 (e) in supernatant were measured by ELISA. Values represented as mean ± SD of at least four independent experiments performed in triplicate. ^#^ *P* < 0.05 compared with the control group, and ^*∗*^ *P* < 0.05 compared with TNF-*α* group. M: morin (10 *μ*M) and T: TNF-*α* (10 ng/mL).

The toxicity of morin (0.1, 1, 5, 10, 50, 100, and 200 *μ*M) to human BECs was first determined. Cell viability was 81%  ± 4% in the 10 *μ*M group at 24 h (Figure 4(a)). BECs have been reported to release chemokines and adhesion molecules to induce an inflammatory response and stimulate eosinophil migration in asthmatic patients [5]. To further ascertain the anti-inflammatory mechanism of morin, we studied its effects on the TNF-*α*-induced expression of proinflammatory proteins in BECs. The results showed that morin (10 *μ*M) dramatically blocked the TNF-*α*-induced upregulation of eotaxin-1, MCP-1, IL-8, and ICAM-1 expression in human BECs (*P* < 0.05) (Figures 4(b)–4(e)).

### 3.8. Morin Diminished TNF-*α*-Induced ROS Generation in Human BECs

**Figure 5:**
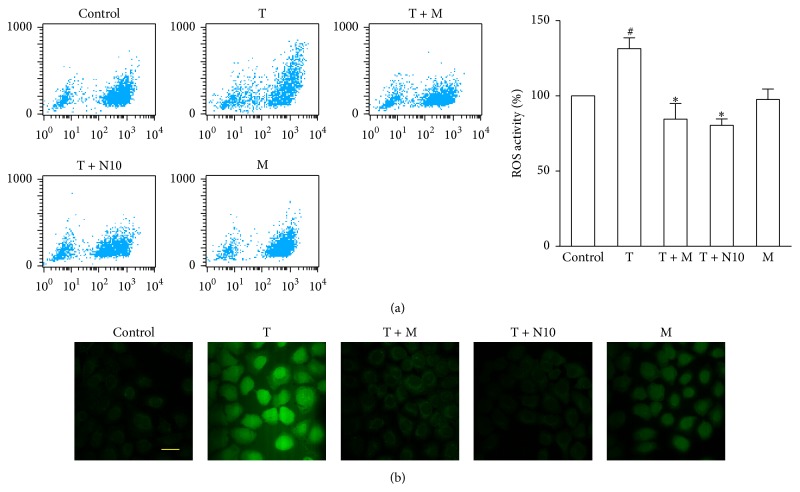
Treatment with morin attenuated intracellular ROS production induced by TNF-*α*. (a) Fluorescence-activated cell sorting profile of ROS generation by flow cytometry. Summary of the average ROS production by BECs treated with morin (10 *μ*M) or NAC (1 and 10 mM) after TNF-*α* (10 ng/mL) stimulation in three independent experiments. (b) DCFH-DA fluorescence (green) imaging of ROS in BECs evaluated with a laser scanning confocal microscope. Scale bar: 50 *μ*m. Values represented as mean ± SD of at least four independent experiments performed in triplicate. ^#^ *P* < 0.05 compared with the control, and ^*∗*^ *P* < 0.05 compared with the TNF-*α* group. M: morin (10 *μ*M); T: TNF-*α* (10 ng/mL); N: NAC.

ROS are considered to mediate the persistent inflammation that occurs in asthma [24]. Therefore, in the present study, we investigated whether it can regulate ROS generation. As shown in Figure 5, ROS production was promoted by TNF-*α*, which is an effective activator in BECs. Flow cytometric analysis showed that pretreatment with morin (10 *μ*M) or NAC (10 mM) decreased the intracellular ROS levels to 85%  ± 18% or 77%  ± 7% (*P* < 0.05), respectively (Figure 5(a)). These significant data revealed that the antioxidant effect of morin may be similar to that of NAC, which is a potent ROS scavenger. In addition, the ROS levels in BECs were monitored using a laser scanning confocal microscope, and similar results were obtained. In brief, we found that morin could apparently suppress TNF-*α*-induced intracellular ROS production in BECs (Figure 5(b)), implying a significant protective effect against oxidative stress.

### 3.9. Morin Suppressed TNF-*α*-Induced MAPK Signaling Activation in Human BECs

**Figure 6:**
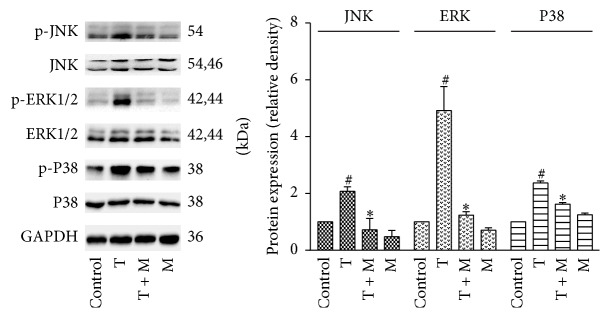
Treatment with morin suppressed the activation of the ERK, JNK, and p38 in BECs. The phosphorylation and total of ERK, JNK, and p38 were measured by western blotting. The relative density quantification is phosphorylated protein relative to total protein. Values represented as mean ± SD of at least four independent experiments performed in triplicate. M: morin (10 *μ*M); T: TNF-*α* (10 ng/mL); ^#^ *P* < 0.05 compared with the control, and ^*∗*^ *P* < 0.05 compared with the TNF-*α* group.

It has been established that MAPK signaling pathways are responsible for oxidative stress-associated airway epithelial damage and that they are crucial for TNF-*α*-induced inflammation in BECs [25]. As shown in Figure 6, we confirmed the effects of TNF-*α* on the activation of ERK, JNK, and p38 in the BECs within 1 h after stimulation and quantified their relative densities (phosphorylated proteins relative to total proteins). This activation was partially blunted in the morin-pretreated group. These results suggested that morin may attenuate TNF-*α*-induced inflammation in BECs by suppressing the activation of MAPK pathways.

## 4. Discussion

Over the last three decades, the prevalence of asthma has markedly increased worldwide. The high costs of asthma treatments pose an immense financial burden to society [1, 26]. Current pharmaceutical options, such as inhaled corticosteroids, long-acting *β* agonists, and other potential agents, have had unsatisfactory effects on controlling asthma. Thus, physicians are searching for novel therapeutic options that are both safe and effective in asthma management [27, 28].

Morin, a natural flavonol, appears to confer a protective effect in chronic inflammatory diseases. In the present study, treatment with morin inhibited the increase of inflammatory cells (including macrophages, eosinophils, and lymphocytes) and downregulated the total IgE, IL-4, and IL-13 levels in OVA-induced mice. Overexpression of IgE and the Th2 cytokines is known to result in eosinophil-rich inflammation, mucus hypersecretion, and enhanced collagen deposition in the lungs [29, 30]. Our findings indicated that morin inhibited inflammatory cell infiltration, mucus hypersecretion, and collagen deposition/fibrosis, implying that it might be valuable as a new antiallergic and anti-inflammatory agent for asthma management.

Several studies have shown that MMP-9, an enzyme that promotes cleavage of the ECM by degrading structural proteins such as collagen, plays a crucial role in the pathogenesis of airway inflammation and remodeling in asthma [31]. The MMP-9 levels in patients with classic asthma are elevated in the serum, sputum, and BALF [32]. MMP-9-deficient animals exhibit reduced airway inflammation, and the immunoreactivity of MMP-9 has been demonstrated to be associated with the severity of asthma [33]. Anti-MMP-9 therapy has been shown to be useful for preventing airway inflammation and remodeling in murine model of asthma [34]. In the current study, we found that morin significantly attenuated MMP-9 expression, which might have contributed to the lessened ECM, thereby contributing to its positive effects on asthma.

Emerging data have provided new insights into the complex interactions that occur between MMP-9 and inflammatory cytokines such as TNF-*α*. The TNF-*α* level is increased in numerous inflammatory diseases, such as rheumatoid arthritis [35], inflammatory bowel disease [36], psoriasis [37], chronic obstructive pulmonary disease [38], and asthma [12]. Evidence obtained from recent studies indicate that the transcriptional and translational activation of MMP-9 is involved in the loss of endothelial barrier integrity induced by TNF-*α* [39, 40]. Airway epithelial injury also leads to an exposure to TNF-*α*, which induces MMP-9 expression, provoking cell migration via various pathways, including the PKC, AP-1, NF-*κ*B, and MAPK pathways [41, 42]. Indeed, anti-TNF-*α* therapy has been reported to reduce the expression of MMP-9 as well as that of other inflammatory cytokines (e.g., IL-4, IL-13, and IgE), thereby hindering the recruitment of inflammatory cells and inhibiting the airway inflammation in asthma [11, 14, 15, 39]. In our study, morin markedly suppressed OVA-induced TNF-*α* and MMP-9 overexpression with attenuation of airway inflammation, implying that its effects might be attributed to the downregulation of TNF-*α*.

Asthma is a pulmonary inflammatory disorder involving excessive oxidative stress. ROS, which are known to contribute to oxidative stress, are primarily produced by eosinophils and other inflammatory cells recruited to the airways in asthma [43–45]. Moreover, stimulated BECs have been shown to generate ROS, exacerbating airway damage, including bronchial hyperreactivity, inflammatory cell infiltration, epithelial cell shedding, goblet cell metaplasia, and mucus hypersecretion [24, 46]. In the present study, severe damage was observed in the airways of the untreated asthmatic mice. Treatment with morin significantly ameliorated these asthma-related pathological injuries. Moreover, the level of MDA, a common indicator of oxidative damage to membrane lipids, in the lung tissues from the OVA-challenged rats was increased, and morin significantly attenuated this increase. This finding revealed that the protective effects of morin in chronic asthma may be partly due to its ROS scavenging activity, resulting in a reduction in OVA-induced oxidative damage.

Accumulating data have shown that ROS are also secondary messengers that are involved in intracellular signal transduction. Increased ROS levels lead to activation of the MAPK pathways [46, 47]. MAPK signaling has been implicated in the transcription of various proinflammatory cytokines (e.g., eotaxin-1, MCP-1, and IL-8) and adhesion molecules (e.g., ICAM-1 and VCAM-1) [48, 49], which contribute to a worsened airway inflammation. It has been well established that eotaxin-1 is important for the delivery of eosinophils to the airways and that it could cause tissue damage and severe inflammation. Many studies have indicated that eotaxin-1 expression is stimulated by TNF-*α* via p38 MAPK/NF-*κ*B signaling [50]. MCP-1 has monocyte and lymphocyte chemotactic activities and stimulates histamine release from basophils. A recent study has confirmed that TNF-*α* induces MCP-1 secretion from human airway smooth muscle cells [51]. IL-8, which is perhaps best known for its proinflammatory effects on immune cells, stimulates the infiltration of neutrophils into the airways in asthma and is associated with severe asthma [52]. ICAM-1 is critical for the transmigration of leukocytes out of blood vessels and into inflamed tissues. Inhibitors of MMPs regulate inflammatory cell migration by reducing ICAM-1 expression in asthma [53]. It has been postulated that TNF-*α* upregulates the production of ROS, which in turn activates BECs to overexpress proinflammatory proteins, such as eotaxin-1, MCP-1, IL-8, and ICAM-1 [24, 54, 55]. These proteins are predominant agents that increase the severity of inflammatory responses. Direct or indirect oxidative stress can also induce BECs to generate TNF-*α* [54, 56]. Thus, a vicious feedback cycle occurs due to the cytotoxic activities of ROS. Our findings showed that morin markedly alleviated the increases in eotaxin-1, MCP-1, IL-8, and ICAM-1 in human BECs, indicating that the relief of airway inflammation might have been due to the downregulation of these proinflammatory proteins, which probably occurred via ROS.

Our findings further demonstrated that morin strongly inhibited the intracellular ROS induced by TNF-*α*, producing effects similar to those of NAC. As a free radical scavenger, NAC prevents oxidant-induced inflammatory mediator release [57]. It is known that ROS and MAPK are both closely related to airway inflammation, but the modulation between them is not clear so far. The previous data confirmed that ERK phosphorylation is ROS-dependent in Siglec-8-mediated eosinophil cell death [58], and JNK phosphatases were critical molecular targets of ROS in TNF-*α*-induced programmed cell death [59]. Pretreatment of cells with the antioxidant enzyme abrogated the thalidomide-induced p38 MAPK activation in adult erythropoiesis [60]. A recent study provided that H_2_O_2_ significantly increased p38 MAPK and ERK1/2 phosphorylation while NAC effectively suppressed phosphorylation of p38 MAPK and ERK1/2 [61]. Additionally, reduction of ROS has been shown to inhibit the TNF-*α*-mediated airway inflammation [62]. These evidences collectively suggested that ROS play key roles in MAPK signaling associated with TNF-*α* stimulation. Therefore, we hypothesize that morin might suppress TNF-*α*-induced inflammation by inhibiting MAPK signaling via ROS.

To confirm this hypothesis, we investigated the effects of morin on the TNF-*α*-induced activation of MAPKs in BECs. The results showed that TNF-*α* induced the phosphorylation of ERK, p38, and JNK in the BECs. Morin pretreatment significantly inhibited the phosphorylation of these kinases, suggesting that morin inhibited TNF-*α*-induced inflammation via the oxidative stress-responsive MAPK pathways.

The present study has confirmed that morin suppresses OVA-induced airway inflammation and ROS as well as inhibiting TNF-*α*-induced ROS/MAPK activation. However, there are some limitations to our study. Although OVA-induced murine models closely mimic human asthma, TNF-*α*, as a proinflammatory cytokine, cannot completely stimulate the development of the complex alterations characteristic of asthma, such as subepithelial fibrosis, airway smooth muscle mass increases (including hypertrophy and hyperplasia), and vascular remodeling. Thus, our findings warrant further evaluations of its* in vitro* and* in vivo* functions as well as its clinical utility in the treatment of delayed allergic diseases.

## 5. Conclusions

In conclusion, we have demonstrated the potential therapeutic action of morin in an experimental model of asthma and its anti-inflammatory properties in human BECs. Collectively, our findings have indicated that morin (I) suppresses both the infiltration of inflammatory cells and the hyperplasia of goblet cells in the airways, (II) reduces MMP-9 expression and fibrosis in OVA-sensitized mice, (III) attenuates elevations in the total IgE, TNF-*α*, IL-4, and IL-13 levels in BALF, and MDA level in lung tissues, (IV) suppresses TNF-*α*-induced eotaxin-1, MCP-1, IL-8, and ICAM-1 expression in human BECs, and (V) inhibits TNF-*α*-induced ROS by regulating MAPK signaling. Taken together, our results provide direct evidence that morin might be a candidate for the adjuvant therapy for asthmatic patients.
